# Feasibility of immersive 40 Hz gamma sensory stimulation in early-stage Alzheimer’s disease

**DOI:** 10.1016/j.tjpad.2026.100679

**Published:** 2026-09-25

**Authors:** Carolina Reis, Laura Hainke, Gabriel Headley, Sergi Navarro, Theodore P. Zanto, Raphael Certain

**Affiliations:** a Clarity Health Technologies, Paris, France; b Department of Neurology, University of California San Francisco, San Francisco, CA, USA; c Neuroscape, University of California San Francisco, San Francisco, CA, USA

**Keywords:** Alzheimer’s disease, Gamma sensory stimulation, Virtual reality, Electroencephalography, 40 Hz, Neuromodulation

## Abstract

Alzheimer’s disease (AD) remains in need of effective therapeutic strategies that are safe, scalable, and broadly accessible. Gamma sensory stimulation (GSS) is a non-invasive neuromodulation approach under investigation as a potential intervention for AD, supported by a favorable safety profile and emerging evidence of biological and clinical effects. This study evaluates the feasibility of delivering immersive GSS through virtual reality (VR) in individuals with early-stage AD.

In this single-session, within-subject, sham-controlled early feasibility study, 11 participants with mild cognitive impairment due to AD or mild AD underwent immersive audiovisual stimulation delivered via VR while electroencephalography (EEG) was recorded. Participants engaged in three VR environments of increasing visual and cognitive complexity, in which audiovisual content was modulated at 40 Hz; two environments also included a sham condition. Primary outcomes were safety, tolerability, and 40 Hz neural responses. Secondary analyses compared neural responses and recall and recognition of encoded video–word pairs between 40 Hz and sham stimulation.

All participants completed the session without serious adverse events or discontinuations. Stimulation-emergent symptoms were predominantly absent or mild. 40 Hz GSS elicited significantly stronger EEG responses at the stimulation frequency than both baseline and sham stimulation (p < 0.001), confirming target engagement. In exploratory analyses, items encoded during 40 Hz stimulation were better recalled and recognized than those encoded during sham stimulation (all FDR-adjusted p < 0.05).

In this single-session study, immersive GSS was well tolerated and elicited frequency-specific 40 Hz EEG responses in individuals with early-stage AD, with no serious safety concerns identified, supporting the feasibility of this delivery approach. The encouraging acute neural and task-specific behavioral differences between 40 Hz and sham stimulation are hypothesis-generating and motivate controlled pilot studies evaluating the effects of repeated use on validated cognitive and functional outcomes.

## Highlights

• Immersive 40 Hz gamma sensory stimulation delivered via virtual reality was safe and well tolerated by individuals with early-stage Alzheimer’s disease.
• Immersive 40 Hz stimulation elicited robust EEG responses at the stimulation frequency relative to baseline and sham conditions.
• In exploratory analyses, recognition and recall performance on the study’s associative-memory task was higher for material presented during 40 Hz audiovisual stimulation than during sham stimulation.

## 1 Background

Alzheimer’s disease (AD) is the leading cause of dementia worldwide and a major contributor to disability and healthcare burden in aging populations. Despite recent therapeutic advances, there remains a need for treatment strategies that are safe, clinically meaningful, scalable, and broadly accessible.

Current standard-of-care approaches include cholinesterase inhibitors and the NMDA receptor antagonist memantine, which may temporarily stabilize cognitive symptoms but do not prevent functional decline and are frequently discontinued. Anti-amyloid monoclonal antibodies, on the other hand, represent a significant scientific milestone as the first disease-modifying therapies approved for AD. However, their clinical effect sizes on cognitive and functional endpoints are modest, and due to safety considerations, eligibility criteria are restrictive, and treatment requires intensive imaging surveillance. Together, these limitations highlight the need for complementary therapeutic approaches.

In this context, gamma sensory stimulation (GSS), typically delivered as rhythmic audiovisual stimulation at 40 Hz, is an emerging non-invasive neuromodulation strategy currently under investigation as a treatment for AD. Gamma oscillations (30–80 Hz) play a central role in coordinating communication between brain regions and supporting cognitive function [1,2] and are disrupted in multiple brain disorders [3], including AD [4,5]. Accordingly, externally driving neural activity at gamma frequencies has been hypothesized to rebalance excitation and inhibition, restore network synchrony, and support cognitive function [[6], [7], [8]].

Beyond its effects on oscillatory dynamics, preclinical studies in AD models suggest that repeated 40 Hz GSS may engage multiple disease-relevant pathways, including neuroinflammatory, glial, and glymphatic processes, and has been associated with reductions in amyloid burden [[9], [10], [11], [12], [13]]. In humans, clinical research on GSS has progressed from early feasibility studies to pivotal evaluation in people with AD. Published studies of repeated daily GSS over several months in individuals with mild-to-moderate AD have reported changes in functional connectivity, inflammatory markers, and measures of brain atrophy, as well as preliminary effects on cognitive and functional outcomes [[14], [15], [16], [17], [18], [19]]. Building on this evidence, GSS is currently being evaluated in the HOPE Study (NCT05637801), a multicenter, randomized, double-blind, sham-controlled pivotal trial of 12 months of daily audiovisual GSS in approximately 670 participants with mild-to-moderate AD across approximately 70 U.S. sites. Its primary endpoint is change in the Integrated Alzheimer’s Disease MMSE–ADCS-ADL Rating Scale (iADMARS), a novel composite of MMSE and ADCS-ADL. The choice of this endpoint may be particularly relevant to GSS because its two component measures showed nominally significant treatment differences in the preceding six-month randomized study [16]. Nevertheless, iADMARS has not undergone the same degree of independent validation as more widely established endpoints such as CDR-SB or iADRS.

Together, the emerging human evidence and ongoing clinical evaluation position GSS as a potential new category of non-invasive, systems-level intervention that may engage multiple disease-relevant pathways and influence network function in AD, with potential for use either as a stand-alone neuromodulation approach or alongside pharmacological strategies targeting specific molecular processes.

While most human implementations of GSS to date have relied on passive stimulation paradigms delivered through simple light flickers alone or paired with modulated sounds, immersive delivery platforms such as VR provide an alternative framework in which rhythmic stimulation can be embedded within structured and interactive environments. Delivering GSS through immersive VR may allow stimulation to be embedded within controlled behavioral contexts, support user engagement during repeated exposure, and enable structured capture of interaction and performance metrics. In a previously reported cohort of cognitively healthy older adults enrolled under the same clinical trial registration, we found that the delivery of immersive 40 Hz GSS via VR was safe, well tolerated, and capable of evoking gamma-band neural responses [20].

Here, we extend this work to individuals with MCI due to AD and mild AD dementia and report findings from a single stimulation session in which GSS was delivered through immersive VR environments while electroencephalography (EEG) was recorded. The primary objectives were to evaluate safety, tolerability, and feasibility of evoking gamma-band neural activity in the patient population. Secondary objectives were to assess differences between 40 Hz and sham stimulation conditions in neural responses and in performance on a study-specific associative-memory task.

## 2 Methods

### 2.1 Study design

This early feasibility clinical study employed a single-session, within-subject, sham-controlled design, in which all participants were exposed to both active and sham stimulation conditions during the same visit. The study was conducted at the Lakeview Institute of Clinical Research (Florida, United States) in accordance with the Declaration of Helsinki and International Council for Harmonisation Good Clinical Practice guidelines. Ethical approval was obtained from the appropriate institutional review board, and all participants or their legally authorized representatives provided written informed consent prior to participation. The protocol was registered at ClinicalTrials.gov (NCT06234930).

### 2.2 Participants

A total of 11 older adults diagnosed with mild cognitive impairment due to AD or mild AD met eligibility criteria and completed the study. There were no dropouts.

Main inclusion criteria were: age 50–90 years; clinical diagnosis of mild AD or mild cognitive impairment due to AD, with the latter confirmed by prior amyloid PET imaging or cerebrospinal fluid biomarkers; Montreal Cognitive Assessment (MoCA) score between 18 and 25 (inclusive); ≥8 years of formal education; fluency in English; and adequate visual and auditory function to complete study procedures. Main exclusion criteria included: prior exposure to monoclonal antibody therapy for AD; recent changes (within 30 days) in memantine or acetylcholinesterase inhibitor treatment; personal or family history of seizure or epilepsy; history of stroke or other significant neurological disorder; migraine; alcohol use disorder within the past two years; major medical conditions that could interfere with study participation; active depression (Geriatric Depression Scale >8); pregnancy; use of hearing aids incompatible with study procedures; or blood-borne pathogens.

### 2.3 Stimulation protocol

Participants completed a single-session protocol in which audiovisual stimulation was delivered during three VR environments that increased in stimulus complexity and level of cognitive engagement: Simple-Passive, Complex-Passive, and Complex-Active. Across environments, synchronized audiovisual stimulation was delivered in intermittent 3-second epochs separated by jittered inter-trial intervals (3.5–4 s). Auditory stimulation remained constant across environments and consisted of an amplitude-modulated pure tone, while visual stimulation varied according to the VR environment. During the 40 Hz condition, auditory and visual signals were synchronously modulated at 40 Hz, whereas sham stimulation used temporally shuffled modulation sequences designed to preserve a similar flicker percept without a spectral peak at 40 Hz (Figure S1).

In the Simple-Passive environment, participants viewed a flickered white panel without performing a task. In the Complex-Passive environment, participants passively viewed flickered naturalistic video clips. In the Complex-Active environment, participants viewed a different set of flickered naturalistic video clips, after which the final video frame remained on screen while a word was displayed to form video–word associations. After every five video–word association trials, a one-minute distractor task was administered in the VR, followed by immediate associative recognition using a three-alternative forced-choice task.

In the Simple environment, participants completed seven blocks of 40 Hz stimulation only. In the Complex environments, participants completed seven blocks per stimulation condition (40 Hz stimulation and sham), with block order randomized. This design resulted in five experimental conditions: Simple-Passive (40 Hz), Complex-Passive (40 Hz), Complex-Passive (sham), Complex-Active (40 Hz), and Complex-Active (sham), each comprising 35 stimulation epochs. See Figures S2 and S3 in the Supplementary Materials for a more detailed description of the experimental paradigm.

### 2.4 Materials

Audiovisual stimulation was delivered while participants were seated using a VR headset (Meta Quest 2) and in-ear headphones (SONY MDR-EX15LP) connected to a desktop computer running custom stimulation software. All stimuli, instructions, and response prompts were presented automatically within the VR environments without manual intervention by study staff. Participants progressed through the VR environments while EEG was recorded and responded verbally to system prompts using integrated voice recognition. These responses included memory task responses during the immediate associative recognition task as well as requests to pause the session if required.

Following completion of the stimulation phase, participants underwent a brief resting-state recording (∼1.5 minutes), after which the headset and EEG equipment were removed. Delayed image recognition and cued word recall for previously encoded video–word associations were subsequently assessed using a separate computer-based interface after an approximately 20-minute delay. Participants then completed the safety and tolerability questionnaire (see Figure S2 in the Supplementary Materials).

### 2.5 EEG acquisition and processing

EEG recordings were obtained during the VR stimulation session using a 64-channel BioSemi ActiveTwo system (BioSemi, Amsterdam, The Netherlands). Electrodes were positioned according to the standard BioSemi 10–20 layout, and five frontal electrodes (AF7, FP1, FPz, FP2, AF8) were removed to accommodate the VR headset. Horizontal electrooculography (EOG) was recorded using two electrodes placed lateral to the eyes. Data were sampled at 2048 Hz.

EEG data were preprocessed using standard procedures implemented in MNE-Python. Continuous data were band-pass filtered (1–100 Hz) and notch filtered at 60 Hz to attenuate line noise. Channels exhibiting excessive noise were identified and interpolated. Ocular artifacts were removed using independent component analysis (ICA). Data were re-referenced to the common average. Data were segmented into epochs time-locked to stimulation onset (–3.5 s to +3.5 s), and epochs containing residual artifacts were excluded based on automated and visual inspection.

Power spectral density (PSD) was computed using Welch’s method in the 1–100 Hz range during baseline (−3 to −0.5 s) and stimulation (0.5 to 3 s) periods, excluding early transient responses. Power at 40 Hz was averaged across a predefined centro-parietal/parieto-occipital region of interest (ROI; FCz, C1, C2, Cz, PO3, PO4, POz, Oz) selected a priori based on prior work [20]. PSD values at each frequency were normalized by the total spectral power within the corresponding epoch (1–100 Hz), yielding relative power values, which were subsequently converted to dB using a log10 transformation. Normalized PSD values at 40 Hz from individual epochs were used for statistical analyses.

### 2.6 Outcome measures

#### 2.6.1 Primary outcomes

Safety

Safety was assessed following completion of the stimulation session using a structured questionnaire capturing potential stimulation-emergent adverse events. Participants rated the presence and severity (none, mild, moderate, severe) of predefined symptoms including general discomfort, headache, tinnitus, eye pain, disorientation, fatigue, nausea, and blurred vision. An open-ended item allowed participants to report any additional discomfort not listed. Serious adverse events and any discontinuation related to adverse effects were recorded.

Tolerability

Tolerability to immersive GSS was assessed using 7-point Likert ratings (1 = not at all tolerable; 4 = neutral; 7 = extremely tolerable) collected for each VR environment (Simple-Passive, Complex-Passive, Complex-Active). Ratings were based on participants’ overall experience of each environment rather than specific stimulation conditions, as questionnaire stimuli were presented on a standard laptop display and did not reproduce the temporal characteristics of 40 Hz or sham stimulation. For each participant, a mean tolerability score across the three VR environments was calculated to reflect overall stimulation tolerability.

Acceptability of VR exposure was assessed using two 7-point Likert items evaluating overall enjoyability (1 = not at all enjoyable; 4 = neutral; 7 = extremely enjoyable) and perceived overwhelm (1 = not at all overwhelming; 4 = neutral; 7 = extremely overwhelming) while looking through the headset. Procedural feasibility was assessed using 7-point Likert ratings evaluating ease of voice-based interaction and clarity of task instructions (1 = very difficult; 4 = neutral; 7 = very easy).

Feasibility

Feasibility of eliciting 40 Hz neural activity was defined as increased 40 Hz power during 40 Hz stimulation periods relative to baseline periods. For primary analyses, 40 Hz stimulation epochs from the three VR environments (Simple-Passive, Complex-Passive, and Complex-Active) were modeled relative to corresponding baseline periods, with subject included as a random intercept.

#### 2.6.2 Secondary outcomes

Secondary neural outcomes

To validate the sham control condition, 40 Hz power during 40 Hz stimulation epochs was compared to sham stimulation epochs within the Complex-Passive and Complex-Active VR environments. Analyses were conducted separately for each environment, comparing 40 Hz and sham stimulation conditions while accounting for subject-level variability.

Secondary behavioral outcomes

Performance on the study-specific associative-memory task was assessed during and after the Complex-Active environment using one immediate and two delayed task measures. Immediate associative recognition was measured as the proportion of correct responses in a three-alternative forced-choice task, in which participants selected the image (last frame of the encoded video) corresponding to each word; responses were coded as binary (correct vs incorrect).

Delayed memory performance was assessed approximately 20 minutes after encoding. Delayed image recognition was quantified using the sensitivity index (d′), calculated from hit and false-alarm rates with log–linear correction for extreme values. Delayed cued word recall was coded as binary (correct vs incorrect) for previously encoded video–word associations.

### 2.7 Statistical analysis

Safety and tolerability outcomes were summarized descriptively. The incidence and severity of stimulation-emergent adverse events were reported as counts and percentages of participants. Overall tolerability ratings were summarized using medians and interquartile ranges.

Statistical analyses were conducted using mixed-effects models to account for the within-subject design. For EEG outcomes, 40 Hz power (PSD) was analyzed using Gaussian linear mixed-effects models, with subject included as a random intercept. The primary analysis compared 40 Hz stimulation periods to baseline periods, with stimulation (40 Hz vs baseline) included as a fixed effect. Secondary analyses compared 40 Hz and sham stimulation in the Complex-Passive and -Active environments, with condition (40 Hz vs sham) included as a fixed effect. For these models, average stimulus brightness was included as a covariate, together with a condition × brightness interaction term to account for potential differences in visual luminance across flickered naturalistic video trials.

Immediate associative recognition and delayed cued word recall were analyzed at the trial level using binomial generalized linear mixed-effects models with condition as a fixed effect and subject as a random intercept. Delayed image recognition sensitivity (d′) was compared between conditions using a paired permutation test. For the three behavioral comparisons, p-values were adjusted using false discovery rate (p_FDR) correction. All tests were two-sided with α = 0.05.

## 3 Results

### 3.1 Participant characteristics

Eleven participants with MCI due to AD or mild AD dementia completed the study (n = 4 and n = 7, respectively). The mean age was 78.7 ± 3.5 years, and 4 participants (36.4%) were female. Ten participants (90.9%) self-identified as White. Participants had a mean of 16.5 ± 2.5 years of education. Mean MoCA score was 21.5 (range: 18–25), consistent with mild cognitive impairment to mild dementia. Mean Geriatric Depression Scale score was 1.6 (range: 0–4), indicating low levels of depressive symptoms.

### 3.2 Safety and tolerability

All 11 participants completed the study session. No serious adverse events were reported, and no participants discontinued the session due to adverse effects. Stimulation-emergent adverse events were predominantly absent or mild (Table 1). The most frequently reported symptom was fatigue, which was rated as mild in three participants (27.3%) and moderate in two participants (18.2%). Headache was reported as mild in one participant (9.1%) and moderate in one participant (9.1%). General discomfort, eye pain, and blurred vision were each reported as mild in one participant (9.1%). No participants reported tinnitus, disorientation, or nausea. No severe adverse events were observed.

**Table 1:** **Adverse events reported after the VR-based stimulation session.** Participants (n = 11) rated predefined symptoms using a 4-point scale (0 = not at all, 1 = mild, 2 = moderate, 3 = severe) in a post-session safety questionnaire. Values represent the number of participants, with percentages in brackets. Blank cells indicate no reported cases. No additional adverse events were reported outside the structured questionnaire.

Adverse Events (n=11)	None n. participants (% participants)	Mild n. participants (% participants)	Moderate n. participants (% participants)	Severe n. participants (% participants)
**General discomfort**	10 (90.9%)	1 (9.1%)	-	-
**Headache**	9 (81.8%)	1 (9.1%)	1 (9.1%)	-
**Tinnitus**	11 (100%)	-	-	-
**Eye pain**	10 (90.9%)	1 (9.1%)	-	-
**Disorientation**	11 (100%)	-	-	-
**Fatigue**	6 (54.5%)	3 (27.3%)	2 (18.2%)	-
**Nausea**	11 (100%)	-	-	-
**Blurred vision**	10 (90.9%)	1 (9.1%)	-	-

Tolerability of flickered stimulation was favorable (participant-level median 5.33 [IQR 4.83–5.83] on a 7-point scale). VR exposure was generally well accepted: six participants rated the experience as enjoyable (>4/7), whereas nine rated it as not overwhelming (<4/7), including seven who rated it as not at all overwhelming (1/7). Voice-based interaction was easy to use, with eight participants providing the maximum rating and none rating below 5/7. All participants rated task instructions as easy to understand (≥5/7).

### 3.3 Neural response to VR-based gamma sensory stimulation

As a key indicator of feasibility, 40 Hz power during stimulation was compared with baseline periods across the three VR environments. A linear mixed-effects model including stimulation (baseline vs 40 Hz stimulation) as a fixed effect and participant as a random intercept revealed a robust increase in 40 Hz power during stimulation (β = 6.50, 95% CI [6.17, 6.82], t(1840) = 39.39, p < 0.001; standardized β = 1.28). The model explained 40% of the variance attributable to fixed effects. As shown in Fig. 1, this effect was characterized by a spatially distributed increase in 40 Hz power across posterior and frontal regions (Fig. 1A), a clear peak at the stimulation frequency in the power spectrum (Fig. 1B), and consistently higher participant-level 40 Hz power during stimulation relative to baseline (Fig. 1C).

**Fig. 1:**
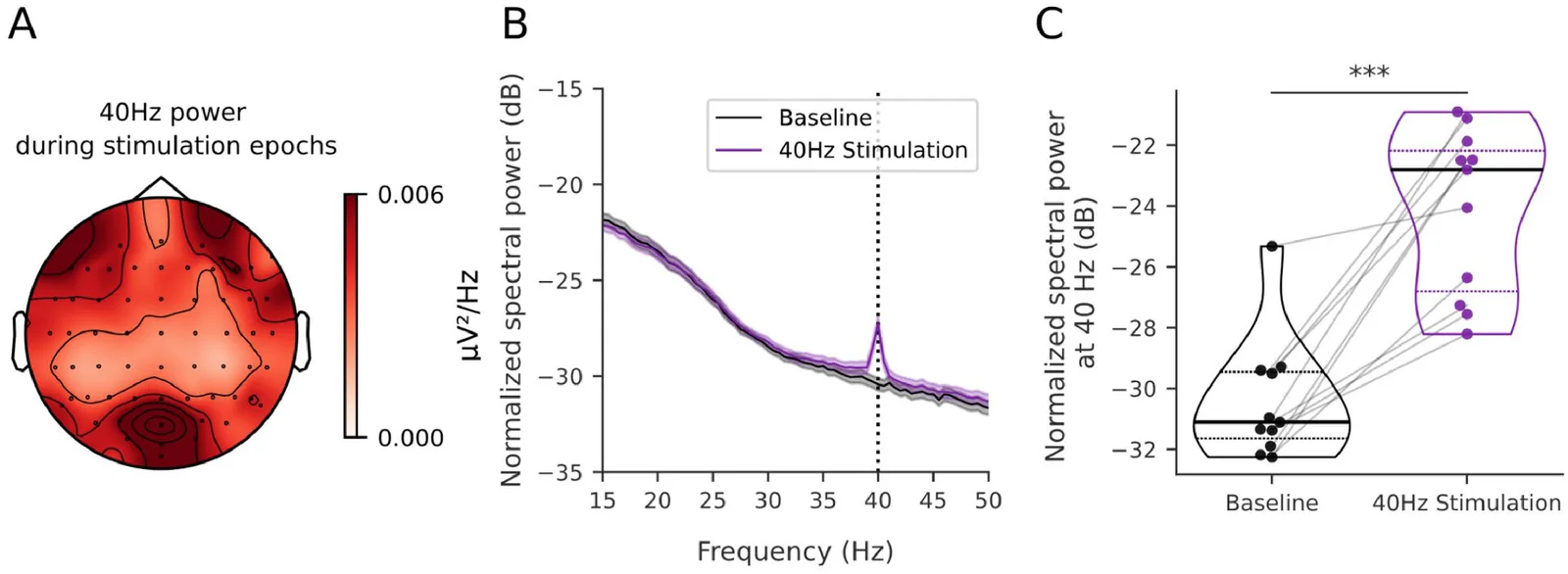
**Neural response at 40 Hz during immersive gamma sensory stimulation.** (A) Scalp topography shows the spatial distribution of normalized 40 Hz power averaged across 40 Hz stimulation epochs pooled across the whole stimulation paradigm (Simple-Passive (40 Hz), Complex-Passive (40 Hz), Complex-Active (40 Hz)). Warmer colors indicate greater power at 40 Hz. (B) Power spectral density averaged across the predefined centro-parietal/parieto-occipital region of interest: during baseline periods (black) and during 40 Hz stimulation periods (purple). The dashed vertical line indicates the stimulation frequency. Shaded areas represent ± SEM across participants. (C) Participant-level normalized spectral power at 40 Hz during baseline and 40 Hz stimulation periods. Each dot represents an individual participant and lines indicate paired observations. ***p < 0.001.

To evaluate the specificity of the neural response and validate the sham control condition, EEG responses during 40 Hz and sham stimulation were compared within the Complex-Passive and Complex-Active environments while accounting for variability in stimulus brightness across naturalistic video stimuli. Power spectral density analyses revealed a clear peak at 40 Hz during active stimulation that was absent during sham stimulation in both environments (Fig. 2A,B).

**Fig. 2:**
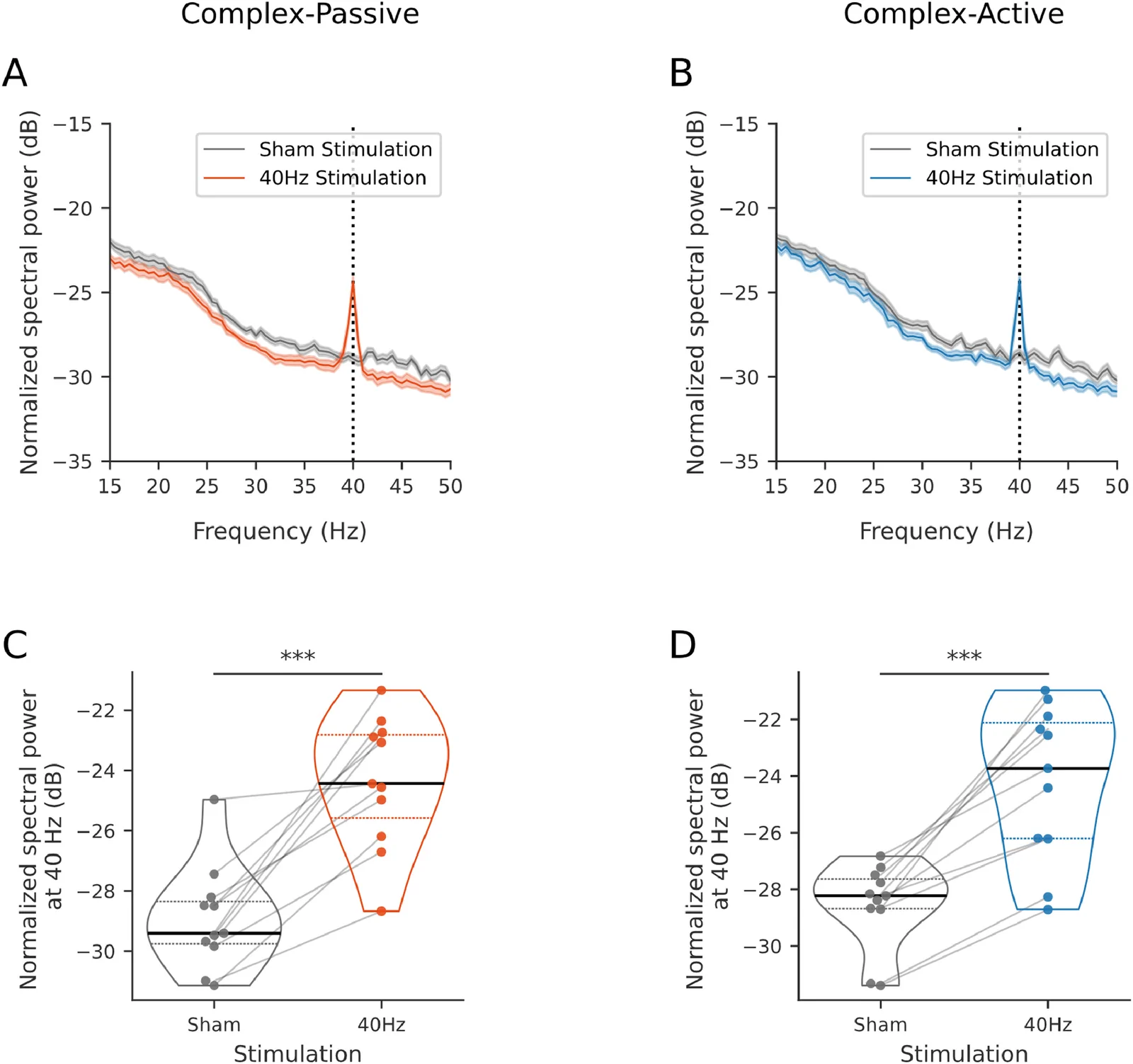
**Neural response at 40 Hz during 40 Hz and sham stimulation.** (A) and (B) Power spectral density averaged across the region of interest during sham and 40 Hz stimulation blocks in the Complex-Passive and Complex-Active environments, respectively. Dashed vertical lines indicate the frequency of interest. Shaded areas represent ± SEM across participants. (C) and (D) Participant-level normalized 40 Hz power during sham and 40 Hz stimulation blocks in the Complex-Passive and Complex-Active environments, respectively; ***p < 0.001.

Consistent with this, 40 Hz power was significantly greater during 40 Hz stimulation compared to sham in both environments. In the Complex-Passive environment, a linear mixed-effects model revealed a significant main effect of condition (β = 3.80, 95% CI [2.26, 5.34], t(625) = 4.85, p < 0.001; standardized β = 1.09), with no significant effects of average stimulus brightness (β = 1.19, p = 0.471) or condition-by-brightness interaction (β = 2.71, p = 0.272) (Fig. 2C). Similarly, in the Complex-Active environment, a significant main effect of condition was observed (β = 3.63, 95% CI [2.43, 4.84], t(609) = 5.91, p < 0.001; standardized β = 1.07), with no significant effects of average stimulus brightness (β = 1.19, p = 0.294) or interaction (β = 2.65, p = 0.135) (Fig. 2D).

### 3.4 Secondary acute behavioral outcomes

Performance on the exploratory behavioral task differed between items encoded during 40 Hz and sham stimulation (Fig. 3). A binomial generalized linear mixed-effects model revealed significantly higher immediate recognition accuracy for video–word pairs encoded during 40 Hz stimulation compared to sham stimulation (β = 0.88, 95% CI [0.44, 1.31], p_FDR < 0.001), corresponding to an odds ratio of 2.41 (Fig. 3A). Delayed image recognition sensitivity (d′) was significantly higher for images corresponding to the last frame of the videos (used as recognition stimuli) encoded during 40 Hz stimulation compared to sham stimulation (mean difference = 0.35, 95% CI [0.21, 0.49], p_FDR = 0.002; Cohen’s d = 1.39) (Fig. 3B). Delayed cued word recall was also significantly greater for items encoded during 40 Hz stimulation relative to sham stimulation (β = 0.44, 95% CI [0.03, 0.84], p_FDR = 0.036; odds ratio = 1.55) (Fig. 3C). The average elapsed time between encoding and delayed testing was 21.23 ± 5.13 minutes (mean ± SD). In sum, performance across all three memory task measures was higher for items encoded during 40 Hz stimulation than for items encoded during sham stimulation. Although encouraging, these acute, condition-specific findings from a single session are not sufficient to establish improvement in cognitive function or therapeutic efficacy.

**Fig. 3:**
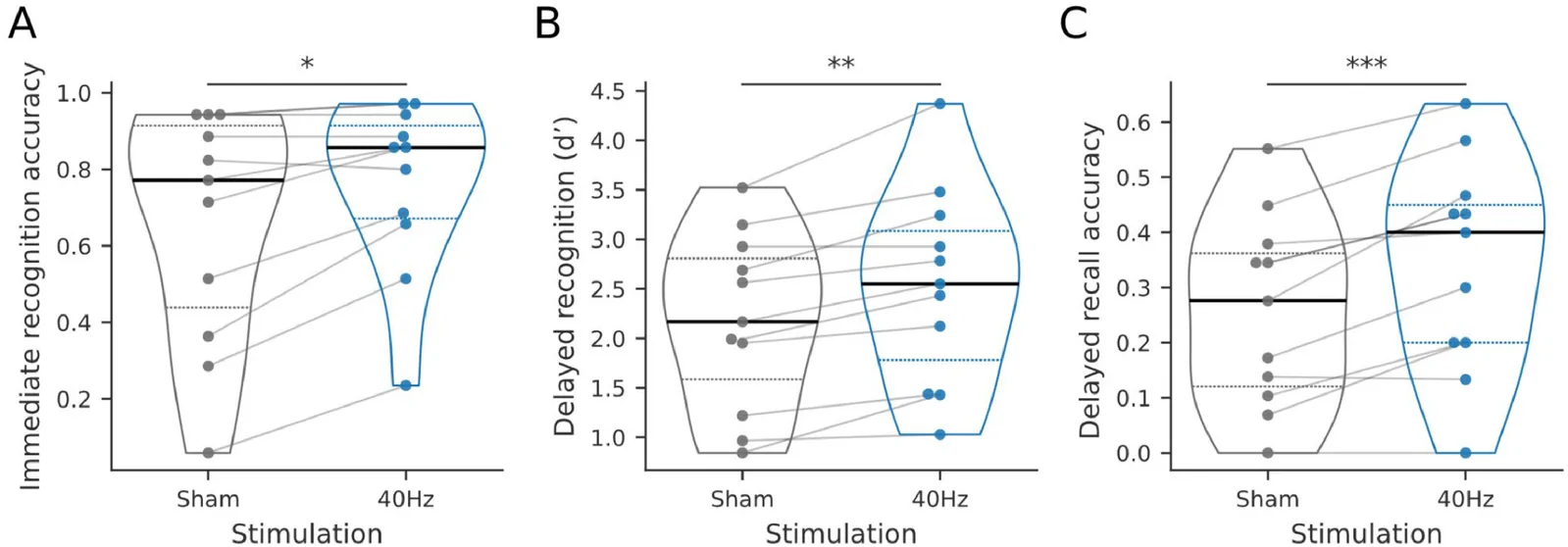
**Secondary behavioral outcomes for video–word associations encoded during sham and 40 Hz stimulation.** (A) Immediate recognition accuracy for video–word pairs encoded during sham and 40 Hz stimulation, assessed using a three-alternative forced-choice task in which participants selected the image (last frame of the encoded video) corresponding to each word. (B) Delayed recognition sensitivity (d′) for images corresponding to video stimuli encoded during sham and 40 Hz stimulation, assessed among a pool of images including foils. (C) Delayed cued recall accuracy for video–word associations encoded during sham and 40 Hz stimulation (word recall given video cue). Dots represent individual participants and lines indicate paired observations across conditions. Violin plots depict the distribution of participant-level values, with solid lines indicating the median and dashed lines indicating quartiles. P-values were corrected for multiple comparisons using the false discovery rate (FDR). *p_FDR < 0.05, **p_FDR < 0.01, ***p_FDR < 0.001.

## 4 Discussion

In this single-session early feasibility study, we evaluated whether immersive 40 Hz audiovisual gamma sensory stimulation delivered through a VR platform was safe, tolerable, and capable of evoking frequency-specific neural activity in individuals with MCI due to AD and mild AD. Three main findings emerged. First, participants completed the session without serious adverse events or discontinuations, and stimulation-emergent symptoms were predominantly absent or mild. Second, the immersive VR environments were generally considered enjoyable, the stimulation embedded within these environments was well tolerated, and the device was easy to interact with. Finally, 40 Hz stimulation robustly increased EEG power at 40 Hz relative to baseline intervals, demonstrating the ability to elicit gamma-band neural activity in an early AD cohort. Furthermore, exploratory analyses showed that 40 Hz stimulation was distinguishable from sham stimulation at both the neural and behavioral levels. Material encoded during 40 Hz stimulation was associated with a distinct 40 Hz power peak and higher recognition and recall scores in the study-specific memory task.

Emerging evidence suggests that 40 Hz gamma sensory stimulation may represent a safe and potentially effective intervention strategy for AD. In the present work, we explored the use of VR technology and its integrated audiovisual and interaction capabilities to deliver 40 Hz stimulation within cognitively engaging environments. Beyond supporting engagement during repeated at-home exposure, VR-based delivery enables simultaneous collection of interaction and cognitive performance measures that may help quantify adherence, behavioral state, and potential stimulation-related effects over time. This may be particularly relevant given evidence that the magnitude of 40 Hz neural responses varies across brain states, with stronger responses observed during wakefulness than during sleep [21]. Together, these features may help reduce and characterize variability in effective stimulation exposure, supporting a more controlled and measurable delivery of the intervention. Although these potential advantages require validation under repeated and remote use conditions, the present study provides an initial evaluation of immersive 40 Hz gamma sensory stimulation in individuals with AD.

The findings of the current study are discussed in turn below. First, our safety findings suggest that immersive VR delivery does not introduce additional acute safety concerns relative to previously reported passive light-and-sound stimulation paradigms [14,16,18,22]. Consistent with findings from cognitively healthy older adults tested using the same platform [20], no VR-associated symptoms commonly linked to motion exposure, such as nausea or disorientation, were reported, either in response to structured queries or in open-ended feedback. Adverse events were predominantly absent or mild and transient. The moderate fatigue reported in two participants and headache in one participant may be attributable to the duration of the VR stimulation session, which lasted approximately 90 minutes including pauses and EEG setup. This duration exceeds the anticipated exposure time of less than one hour planned for future studies. While the present study was limited to a single supervised session in a small sample, these findings suggest that embedding rhythmic stimulation within immersive environments does not appear to alter the acute safety profile observed with non-immersive implementations.

Participants were able to engage with the VR-based platform under supervised conditions, with favorable ratings for usability and instruction clarity. Most participants did not perceive the VR experience as overwhelming and reported neutral-to-positive enjoyment ratings. These findings are consistent with prior literature indicating that older adults, including those with cognitive impairment, can tolerate structured VR-based interventions when appropriately designed [[23], [24], [25]]. Nonetheless, usability, adherence, and sustained engagement under repeated and unsupervised conditions will require formal evaluation before conclusions can be drawn regarding long-term feasibility.

Consistent with prior reports, immersive 40 Hz GSS robustly increased neural oscillatory power at the stimulation frequency (40 Hz) in individuals with early-stage AD, with a fronto-occipital scalp distribution [18,[26], [27], [28]]. These findings further support the feasibility of delivering GSS via immersive VR and demonstrate that gamma-band neural responses can be reliably elicited across visual environments differing in sensory complexity, cognitive load, and interactivity demands [20].

Importantly, the EEG analyses employed here are limited to cortically evoked activity and cannot determine engagement of deeper structures. Nonetheless, prior work suggests that gamma-frequency sensory stimulation can propagate beyond primary sensory regions and that the magnitude and spatial distribution of gamma responses may be influenced by cognitively relevant content and task demands [27,29,30]. For example, high-density EEG studies using gamma-modulated audiovisual stimuli during memory paradigms have reported hippocampal-localized phase-locked activity associated with successful encoding [29], while intracranial and network-level studies further suggest that cognitive context may shape the propagation of gamma responses across distributed brain regions [27,30]. Together, these findings support future mechanistic investigations using intracranial or high-density EEG approaches to examine whether task-integrated VR-based GSS differentially engages brain networks vulnerable in AD, including those involving the hippocampus.

Across immediate recognition, delayed cued word recall, and delayed image recognition, memory performance in the study-specific memory task was higher for items encoded during active 40 Hz stimulation than for items encoded during sham stimulation. These measures sample associative encoding, item memory, and recall processes that depend, at least in part, on medial temporal lobe function, which is particularly vulnerable in AD. While these findings are in line with prior longitudinal studies in which associative memory performance was modulated following repeated passive GSS [14] or 40 Hz transcranial alternating current stimulation (tACS) delivered over several weeks to months [31], comparisons must be made cautiously because of differences in stimulation modality or delivery mode, treatment duration, outcome measures, and study design. These analyses were exploratory and based on a single session in a small cohort; they therefore do not establish improvement in cognitive function. Rather, these encouraging, task-specific findings are hypothesis-generating and support stepwise, controlled pilot studies of repeated use — focused first on safety and validated cognitive outcomes — before larger therapeutic trials.

Finally, in contrast to passive stimulation paradigms with comparatively constrained sensory input, immersive and task-based VR environments introduce additional sensory and cognitive features, making it essential to isolate the specific effects of 40 Hz stimulation from these non-specific influences. In this context, the implementation of a sham condition that did not contain a 40 Hz peak at the stimulus level (Fig. S1), and was distinguishable from active 40 Hz stimulation at the neural level (Fig. 2), strengthens the internal validity of the comparison between active and sham conditions. Although a technically robust sham is only one component of readiness for larger randomized therapeutic trials, which will also require evidence of longer-term safety, sustained effects on validated outcomes, and reproducibility, its successful implementation provides an important methodological foundation for controlled longitudinal evaluation of immersive GSS.

### 4.1 Conclusion

Taken together, findings from this single-session study suggest that 40 Hz gamma sensory stimulation delivered within cognitively engaging, interactive, and immersive environments is a safe and well-tolerated neuromodulation approach capable of eliciting gamma-band neural responses in individuals with early AD under supervised conditions. Differentiation between active and sham stimulation at the neural level, together with high usability ratings, supports further evaluation in controlled longitudinal pilot studies. Accordingly, future studies should evaluate repeated at-home delivery of immersive 40 Hz GSS, with a focus on safety, adherence, and effects on validated cognitive and functional outcomes in AD over extended periods.

## Declaration of generative AI and AI-assisted technologies in the manuscript preparation process

During the preparation of this manuscript, the authors used ChatGPT (OpenAI) to assist with language editing and manuscript refinement. The authors reviewed and edited all content and take full responsibility for the content of the published article.

## Funding information

This study was funded by Clarity Health Technologies.

## Data statement

The de-identified data that support the findings of this study are available from the corresponding author upon reasonable request, subject to applicable ethical, privacy, and contractual restrictions.

## CRediT authorship contribution statement

**Carolina Reis:** Writing – original draft, Supervision, Resources, Project administration, Methodology, Investigation, Conceptualization. **Laura Hainke:** Visualization, Formal analysis, Data curation. **Gabriel Headley:** Software, Investigation, Data curation. **Sergi Navarro:** Resources, Project administration, Investigation. **Theodore P. Zanto:** Writing – review & editing, Validation. **Raphael Certain:** Funding acquisition.

## Declaration of interests

The authors declare the following financial interests/personal relationships which may be considered as potential competing interests: Carolina Reis has patents pending to Clarity Health Technologies. Raphael Certain has patents pending to Clarity Health Technologies. Gabriel Headley has patents pending to Clarity Health Technologies. The corresponding author is co-founder and Chief Scientific Officer of Clarity Health Technologies. Several co-authors are employees, advisors, equity holders, and/or inventors on intellectual property related to the technology described in this manuscript. These relationships are fully disclosed in the manuscript’s Conflicts of Interest statement. If there are other authors, they declare that they have no known competing financial interests or personal relationships that could have appeared to influence the work reported in this paper.
