# Supplementary material for: Feasibility of immersive 40 Hz gamma sensory stimulation in early-stage Alzheimer’s disease
Source: J Prev Alzheimers Dis. 2026 Sep 25;13(10):100679. doi: 10.1016/j.tjpad.2026.100679 (PMC13635571; doi:10.1016/j.tjpad.2026.100679)
Supplement: Supplementary file 1 [file mmc1.pdf]

Feasibility of Immersive 40 Hz Gamma Sensory Stimulation in Early-stage Alzheimer's Disease

Supplementary Materials

Virtual Reality as an audiovisual platform

In the present study, sensory stimulation was delivered using a virtual reality (VR) system comprising a head-mounted display (Meta Quest 2) and in-ear headphones. A head-mounted display is a self-contained headset worn over the eyes, with internal high-resolution displays viewed through lenses that fill most of the visual field and limit competing external visual input, creating a sense of immersion in the presented environment. Functionally, the system operates like a conventional screen and audio device: the headset presents static images or video, while the headphones deliver accompanying sound. However, the enclosed display provides more standardized viewing conditions across participants by maintaining a consistent viewing geometry and minimizing the influence of ambient light. The brightness of the visual content and the amplitude of the accompanying sound could therefore be precisely and synchronously modulated—here, at 40 Hz—to deliver combined audiovisual gamma sensory stimulation. We verified that the intended modulation was faithfully reproduced by the VR system, with stable temporal modulation and high audiovisual synchrony, as reported in Figure S1 and Table S1. Beyond stimulation delivery, the immersive and self-contained platform may offer practical advantages for GSS, including standardized and engaging exposure and a portable format potentially suitable for scalable, home-based use.

Audiovisual Signal Validation

Sound output and photodiode recordings were acquired before and after each participant stimulation session to verify accurate temporal modulation at 40 Hz during active stimulation and the absence of a spectral peak at 40 Hz during sham stimulation (Figure S1). Additional validation metrics describing the temporal stability and synchrony of audiovisual stimulation delivered via the VR platform are summarized in Table S1.

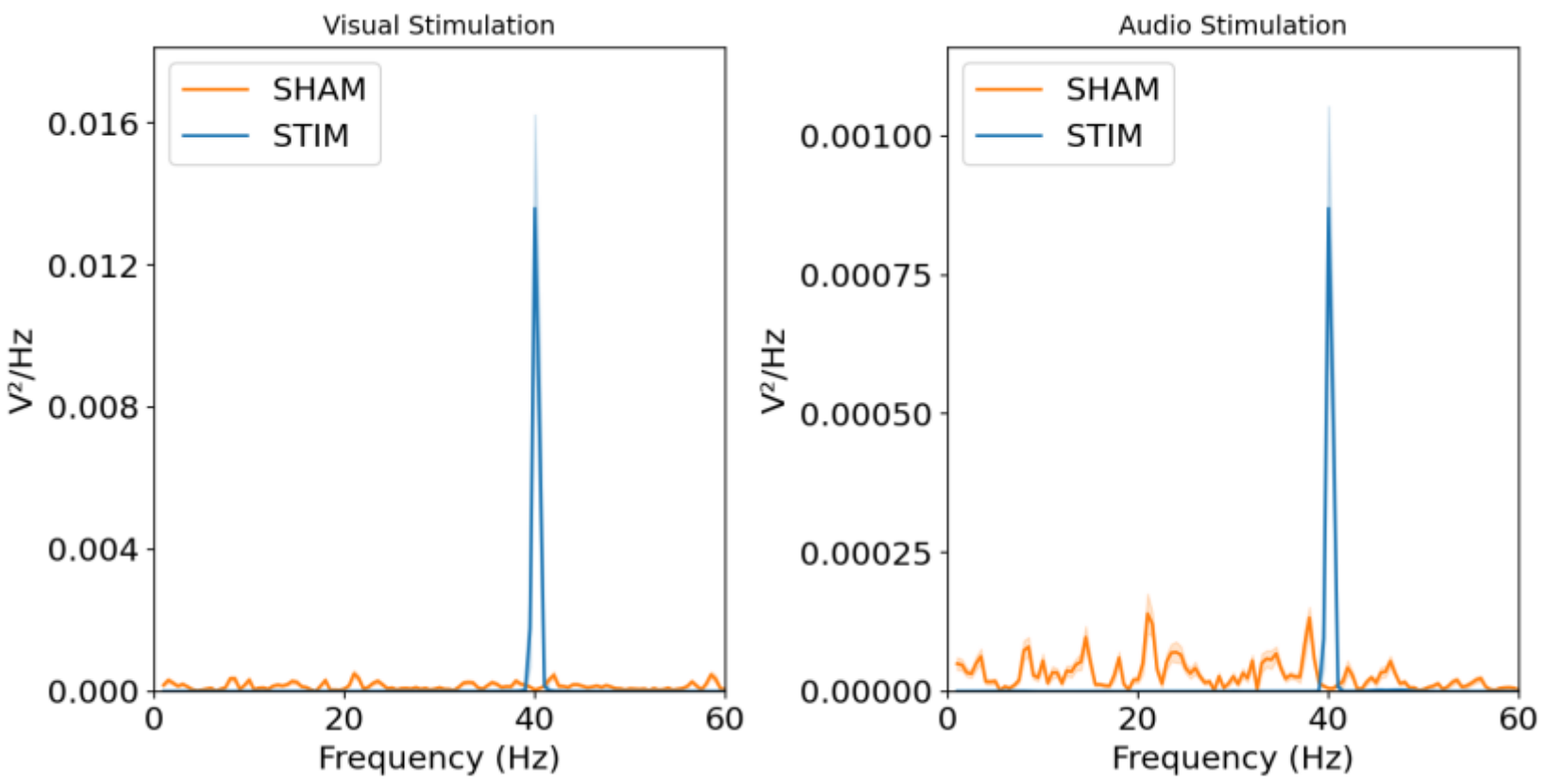

Figure S1 - Spectral validation of audiovisual stimulation signals. Power spectral density of the visual stimulus (left; photodiode recordings) and auditory stimulus (right; audio output

recordings), confirming the presence of a 40 Hz spectral peak during active stimulation and the absence of a periodic component at 40 Hz during sham stimulation.

Mean ± SD	MaxPeak Freq (Hz)	MaxPeak Power (a.u.)	Inter-pulse interval (ms)	Jitter (ms)	PLV
Visual stimulus	40.0 ± 0.00	0.28 ± 0.02	24.9 ± 0.00	1.3 ± 0.7	0.95 ± 0.04
Auditory stimulus	40.0 ± 0.04	0.26 ± 0.02	24.9 ± 0.00	0.3 ± 0.1	0.99 ± 0.01

Table S1 - Validation metrics of audiovisual stimulation signals. For each participant, peak frequency, spectral peak magnitude, inter-pulse interval (IPI), jitter, and phase-locking value (PLV) were computed from photodiode (visual stimulus) and audio output (auditory stimulus) recordings. Metrics were computed across 15 stimulation trials for pre- and post-session recordings and averaged to obtain a single subject-level estimate. Group values represent mean ± SD across participants (n = 11). The mean audiovisual onset asynchrony (SOA) was 4 ± 8 ms, and the phase-locking value between visual and auditory signals was 0.94 ± 0.05. No systematic differences were observed between pre- and post-session recordings for spectral peak frequency, spectral magnitude, inter-pulse interval, jitter, PLV, or audiovisual delay. These measurements confirm stable temporal modulation and synchronization of visual and auditory stimulation signals at the target frequency of 40 Hz during active stimulation.

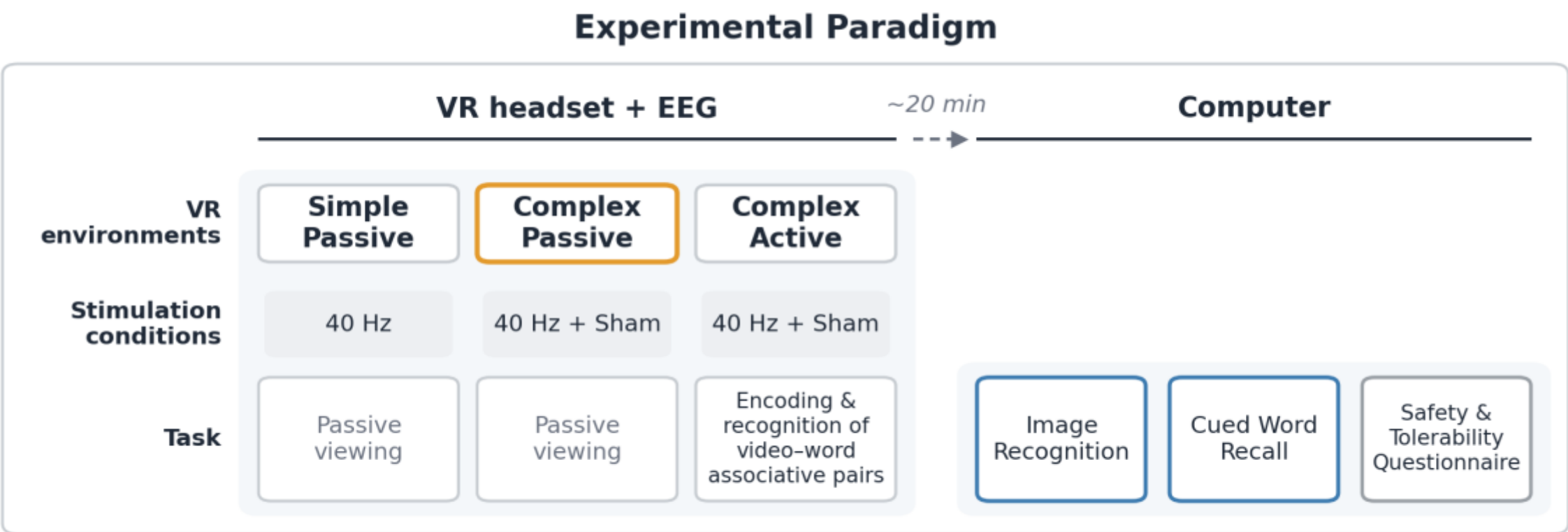

Figure S2 - Experimental Paradigm. Participants completed a single-session protocol in which immersive audiovisual stimulation was delivered across three VR environments of increasing visual and cognitive complexity: Simple-Passive, Complex-Passive, and Complex-Active. Across environments, stimulation was delivered in intermittent 3-second epochs separated by jittered inter-trial intervals (3.5–4 s). The order of VR environments was fixed across participants to ensure a consistent temporal structure between encoding during the Complex-Active condition and subsequent delayed memory assessments, while also supporting gradual familiarization with the VR experience. Because VR environments were presented in a fixed order, environment-specific effects cannot be dissociated from potential effects of time-on-task, fatigue, or familiarization. Within the Simple-Passive environment, participants were exposed to 40 Hz stimulation only. In the Complex-Passive and Complex-Active environments, participants were exposed to both 40 Hz

and sham stimulation conditions, with block order randomized within each environment. Participants completed seven blocks per condition, corresponding to 35 stimulation epochs per experimental condition. The Complex-Active environment included an associative memory encoding task in which participants formed video–word associations during stimulation. Immediate associative recognition was assessed intermittently during the session. Following completion of the VR phase and removal of the headset and EEG equipment, delayed memory performance was assessed outside of VR using image recognition and cued word recall tasks after an approximately 20-minute delay. Safety and tolerability were evaluated at the end of the session.

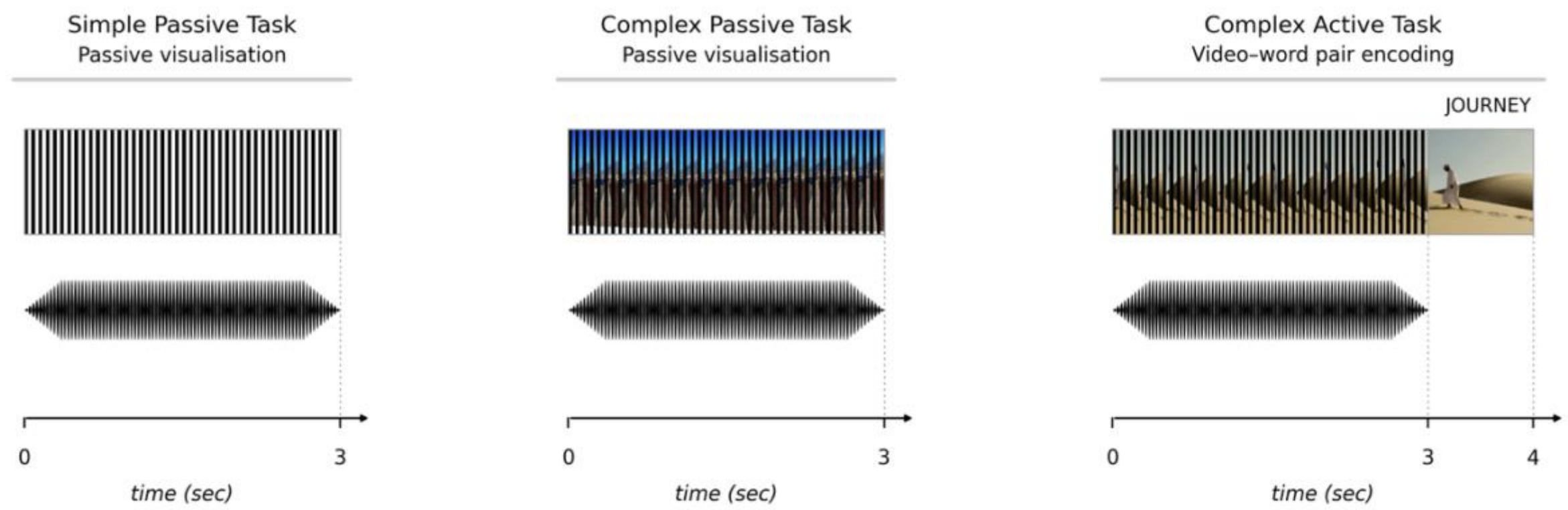

Figure S3 – Illustrative example of a single trial in each VR environment. The images shown are for illustrative purposes only and do not depict the actual visual content presented to participants. The figure shows a representative single trial from each of the three VR environments of increasing visual and cognitive complexity (Simple-Passive, Complex-Passive, and Complex-Active; see Figure S2). During each 3-second stimulation epoch, the brightness of the visual content and the amplitude of the accompanying sound were synchronously modulated at 40 Hz, represented by the vertical bars (visual luminance modulation) and the lower waveform (audio amplitude envelope). In the Simple-Passive environment, stimulation was delivered over a uniform white visual field; in the Complex-Passive and Complex-Active environments, stimulation was applied to naturalistic video content. In the Complex-Active environment, each video was paired with a written word (e.g., "JOURNEY") to form the video–word association assessed in the associative memory task. Flicker bars are shown for illustration and are not drawn to scale (actual modulation frequency, 40 Hz).
